# The COMmunity of Practice And Safety Support (COMPASS) total worker health™ study among home care workers: study protocol for a randomized controlled trial

**DOI:** 10.1186/1745-6215-15-411

**Published:** 2014-10-27

**Authors:** Ryan Olson, Diane Elliot, Jennifer Hess, Sharon Thompson, Kristy Luther, Brad Wipfli, Robert Wright, Annie Mancini Buckmaster

**Affiliations:** Oregon Institute of Occupational Health Sciences, Oregon Health & Science University, 3181 SW Sam Jackson Park Road, L606, Portland, OR 97239-3098 USA; Department of Public Health & Preventive Medicine, Oregon Health & Science University, 3181 SW Sam Jackson Park Road, CB 669, Portland, OR 97239-3098 USA; Department of Psychology, Portland State University, P.O. Box 751, Portland, OR 97207-0751 USA; Division of Health Promotion & Sports Medicine, Oregon Health & Science University, 3181 SW Sam Jackson Park Road, L606, Portland, OR 97239-3098 USA; Labor Education & Research Center, University of Oregon, 1675 Agate Street, Eugene, OR 97403-1289 USA; Department of Psychology, Brigham Young University – Idaho, 525 South Center Street, Rexburg, ID 83460 USA

**Keywords:** Home care workers, Total worker health, Occupational safety, Health promotion, Ergonomics

## Abstract

**Background:**

Home care workers are a high-risk group for injury and illness. Their unique work structure presents challenges to delivering a program to enhance their health and safety. No randomized controlled trials have assessed the impact of a Total Worker Health™ program designed for their needs.

**Methods/design:**

The COMPASS (COMmunity of Practice And Safety Support) study is a cluster randomized trial being implemented among Oregon’s unionized home care workers. Partnering with the Oregon Home Care Commission allowed recruiting 10 pairs of home care worker groups with 8 participants per group (n = 160) for balanced randomization of groups to intervention and control conditions. Physiologic and survey evaluation of all participants will be at enrollment, 6 months and 12 months. Primary outcomes are to increase health promoting (for example, healthy nutrition and regular physical activity) and health protecting (that is, safety) behaviors. In addition to assessing outcomes adjusted for the hierarchical design, mediation analyses will be used to deconstruct and confirm the program’s theoretical underpinnings and intervention processes. Intervention groups will participate in a series of monthly 2-hour meetings designed as ritualized, scripted peer-led sessions to increase knowledge, practice skills and build support for healthy actions. Self-monitoring and individual and team level goals are included to augment change. Because generalizability, reach and achieving dissemination are priorities, following initial wave findings, a second wave of COMPASS groups will be recruited and enrolled with tailoring of the program to align with existing Home Care Commission educational offerings. Outcomes, process and mediation of those tailored groups will be compared with the original wave’s findings.

**Discussion:**

The COMPASS trial will assess a novel program to enhance the safety and health of a vulnerable, rapidly expanding group of isolated caregivers, whose critical work allows independent living of frail seniors and the disabled.

**Trial registration:**

ClinicalTrials.gov identifier: NCT02113371, first registered 11 March 2014.

## Background

The National Institute of Occupational Safety and Health (NIOSH) advocates Total Worker Health™ (TWH), which integrates occupational safety with health promotion as an effective and efficient means to achieve worker well-being [1, 2]. Comprehensive TWH programs combine efforts to reduce job-related injuries and illnesses, while also promoting and protecting employee health. NIOSH has funded four TWH Centers (http://www.cdc.gov/niosh/twh/centers.html), and this intervention protocol is a NIOSH-funded project of the Oregon Healthy Workforce Center of Excellence.

The project targets home care workers (HCWs), a vulnerable occupational group whose work structure presents unique challenges when designing a health protection and health promotion program. HCWs generally are low-wage, unskilled women who care for frail seniors and people with disabilities, by assisting with self-care activities (bathing, eating, personal hygiene, and transferring) and self-management tasks, such as housekeeping, meal preparation, and transportation. These critical services allow clients to remain in the community instead of needing institutionalized care. Although HCWs are the most rapidly expanding sector of the health care industry, with numbers expected to increase 50% by 2022 [3], their isolating work in private homes makes their two million members largely invisible.

HCWs are a group at high risk for occupational injuries [4]. They are without typical occupational safety support structures, such as safety committees, environmental cues for safe behaviors, and co-worker and supervisor assistance. They often lack basic protective equipment, such as gloves and aprons [5], and they frequently perform physically demanding tasks without co-worker assistance or equipment to reduce ergonomic exposures, resulting in high injury rates [6–10]. Health promotion is especially important, as the lower socioeconomic status of HCWs places them at increased risk for health problems [11], and some health problems, such as higher prevalence of obesity [12], also impact their injury rates [13].

As a group, HCWs are characterized as intrinsically caring individuals [14] and, although they often bond with clients and are appreciated by them and their families, those relationships also can be stressful; for example, clients may have progressive illnesses and be nearing the end of life. Moreover, an additional psychosocial challenge is the dual-relationships of clients being both the care recipient and “boss,” who can terminate employment. The boundaries of care can blur, with clients expecting or asking for services that are not on their approved care plan. That ambiguity may pressure HCWs to suppress their own emotions or perform tasks outside their roles to please their clients, again without the immediate support of co-workers or supervisors [15].

### Oregon’s unionized home care workers

Oregon is a leader in advancing the needs of HCWs. It was one of the first US states where HCWs unionized; a situation now present in almost a dozen states. Ability to collectively bargain led to basic benefits, such as health insurance, and establishment of an Oregon Home Care Commission and its registry to match HCWs with consumer employers [16]. Unlike HCWs employed by a private agency, this subset of unionized HCWs identifies potential consumer-employers through the registry and negotiates care plans with assistance of a case manager.

Although unionization secured collective bargaining and health insurance benefits, it did not result in a significant increase in pay, and it has not changed HCW demographics. To become a HCW, the Oregon Home Care Commission only requires being 18 years old, passing a criminal background check, completing an orientation, and providing references to consumer-employers. More than 17,000 HCWs, about two-thirds of whom are currently working, are listed with the registry, and approximately 1,100 Oregonians looking to hire help consult the website each month [17].

Oregon’s system of home care offers a unique opportunity to assess a HCW TWH program. The large number of independent contractors within a single system allowed forming HCW groups and randomizing those groups to condition. Working with private agencies would have been more difficult. Randomizing within agencies would mean potential diffusion to the control condition, as workers often cross-cover clients, and recruiting enough agencies, each with its own educational programs and resources, and to use that as a unit of randomization would have been more costly and generated a sample with greater heterogeneity across groups.

### Aims

Our overarching objective is to create a feasible, exportable TWH program for HCWs that provides knowledge, skills, and social support to enhance their safety and health. The intervention was titled the COMPASS (COMmunity Of Practice And Safety Support) Program. We hypothesized that the intervention group would demonstrate beneficial effects related to primary outcomes of health promotion (healthy nutrition, regular physical activity) and health protection (correcting hazards in homes and safety behaviors).

### Theoretical basis and intervention format rationale

**Figure 1:**
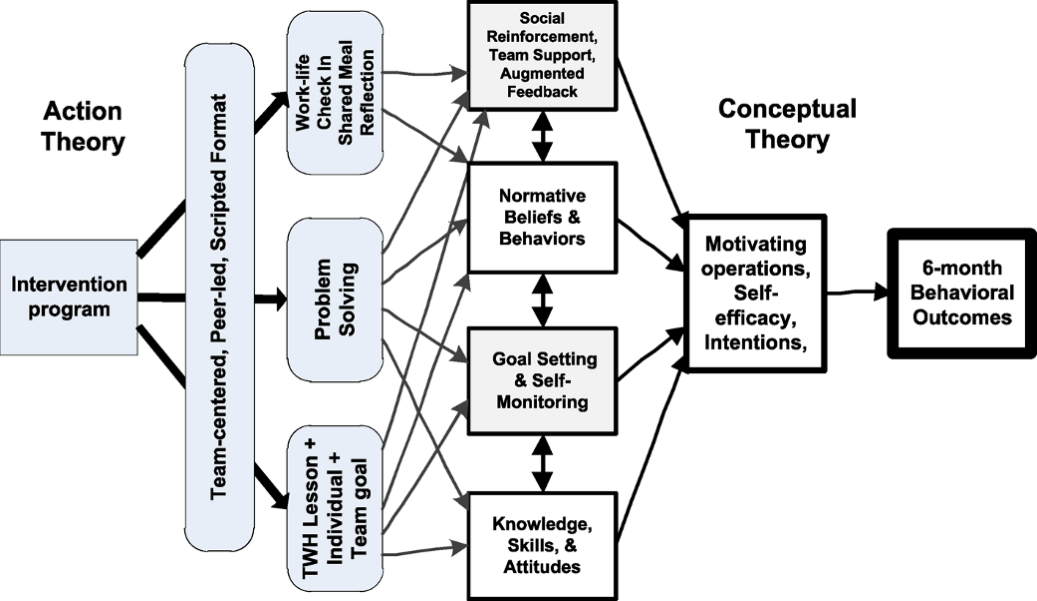
**Conceptual and action theory of the COMPASS Program.** `COMPASS, COMmunity of Practice And Safety Support; TWH, Total Worker Health™.

Even the simplest intervention does not directly change behavior, but depends on altering intermediate or proximal mediating variables. Accordingly, the intervention process can be deconstructed into a conceptual theory that links those proximal variables to desired behavioral outcomes and an action theory that connects the intervention to those same proximal variables [18]. The conceptual aspect is based on behavioral theories and empirical relationships among antecedent variables and intended outcomes. The action theory relates to how the intervention is structured to impact those proximal variables [19]. Our simplified action and conceptual model is shown in Figure 1.

#### Conceptual theory

The conceptual underpinnings of COMPASS are based on social cognitive [20, 21] and reinforcement theory [22], which emphasize a dynamic interaction among three domains: 1) the environmental; 2) an individual’s internal determinants, such as personal motivation, beliefs, self-efficacy and outcome expectancies for different actions; and 3) an individual’s behaviors, which for our purposes relate to health protection and promotion. Although not often mentioned, an individual’s knowledge also relates to the change processes, as it is integral to perceived susceptibility, norms, and expectancies. While conventional thought is that knowledge alone does not change behavior, it may have an important permissive role, as interventions relying on social support are more effective when education is included. For example, in a meta-analysis of studies with unpaid family caregivers, those that combined education with social support produced the largest effects for improved well-being [23].

Given the isolation of home care work, our design also was informed by Bandura’s elaborated Social Cognitive Theory of Self-Regulation [24]. In this theory, discrepancies between one’s current behavior and a personal or social standard are motivating, and behaviors that reduce that gap are reinforcing and enhance self-efficacy [25].

#### Action theory

The COMPASS intervention’s action theory relates to building cohesive HCW groups as vehicles to affect knowledge, skills, norms, social support and self-monitoring elated to TWH behaviors. Our objective was to create bonded teams that could function as “communities of practice”. A community of practice is characterized by a social network of members who share work-related abilities and experiences and use their combined expertise to solve problems, provide support, and advance their profession [26–28]. Originally described for midwives, meat cutters, and tailors, the “community” is the structure that facilitates the interactions, and the “practice” is the shared knowledge and professional identity [28].

The intervention emphasizes goal setting and behavioral self-monitoring to identify discrepancies, a process shown to alter workplace safety behaviors [29]. This also fits well with Social Cognitive Theory’s emphasis that monitoring progress towards objectives affects self-efficacy [24, 30]. Finally, we also sought to integrate goal setting and self-monitoring processes at an individual and group level. Although individual goals and rewards are important, they lack a focus on shared accomplishments and group accountability. Therefore, we supplemented individual goals with group goals, where attainment required the majority of members reach an interdependent target [31]. Having these two levels (individual and group goals) operating simultaneously can be synergistic and augment productivity [32].

Individually recruited HCWs were assigned to a group, with intervention groups meeting serially over several months. The initial meeting is 4 hours, followed by a series of monthly 2-hour meetings. That timing and duration were considered most feasible based on conversations with HCWs during preliminary study of their work activities [7, 8]. To facilitate commitment, the initial meeting was longer, involved more team building activities and transitioned from an investigator to peer-led format. The structure of the monthly meetings was designed to be ritualized, peer-led, and guided by an easy to follow, explicitly scripted team leader manual and corresponding workbooks.

The meetings were designed with four components. They begin with a 10-minute work-life check-in, where each participant writes two ratings on a white board, one for their work and one for their life, using a 1 = worst ever to 10 = best ever scale. The activity is part of the structure developed for the Ignatian Faculty Forum [33, 34], where it is used to acknowledge the importance of each member and provide a context that supports the whole person. Individuals share a few sentences about their ranking in each category before proceeding with the education and social support activities. Rather than engage in discussion, others are encouraged to acknowledge each person’s ratings and events.

**Figure 2:**
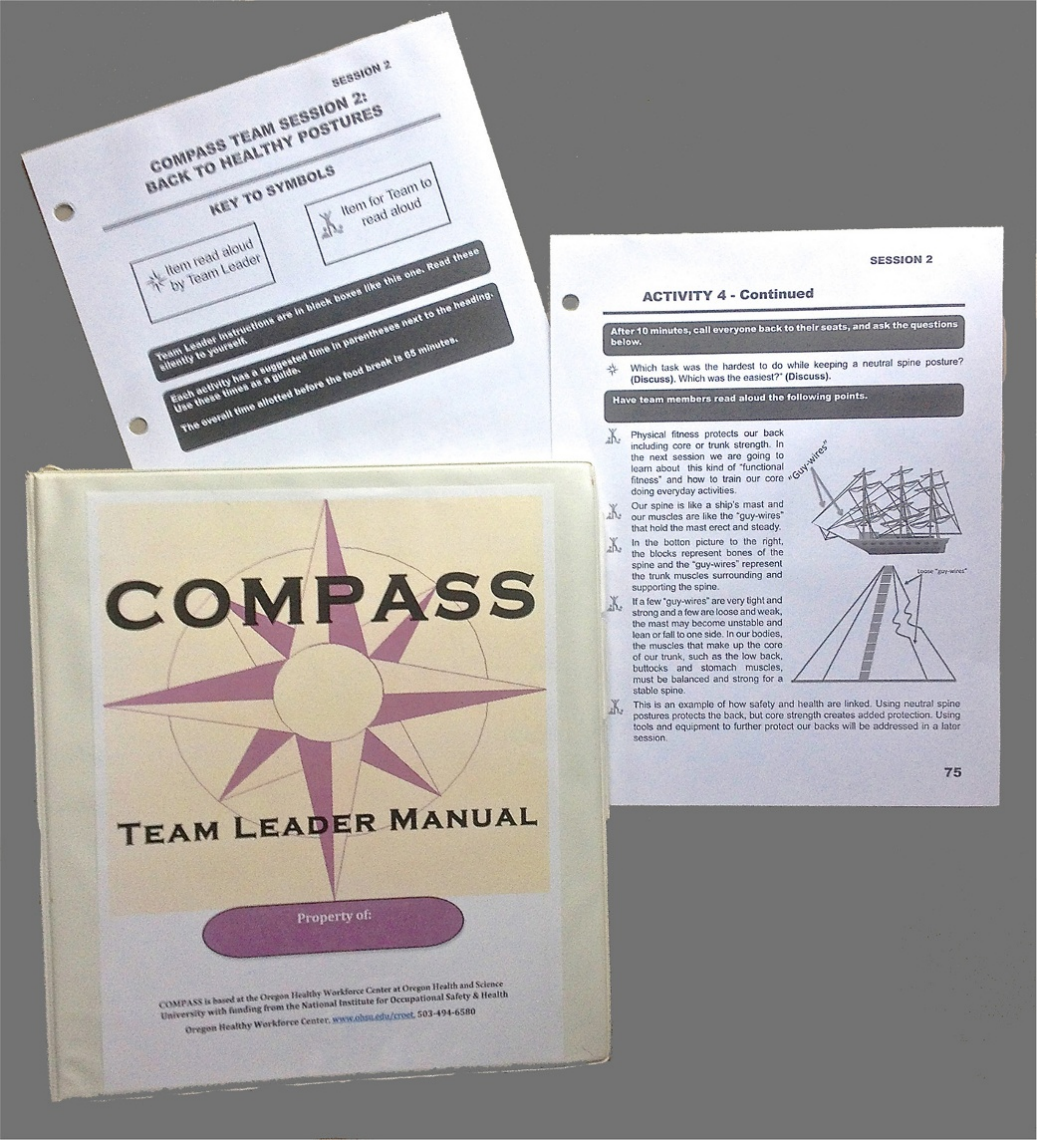
**COMPASS team leader manual and sample scripted pages.** COMPASS, COMmunity of Practice And Safety Support.

Modeled on our prior scripted team-based programs [35, 36], the meetings are led by one of the HCWs in the group. Team leaders were recruited based on their interest during enrollment. Prior to the first team meeting, they participated in a 3-hour team leader training, where they received additional project background and practiced with the scripted format. Examples of the scripted COMPASS lesson plans are presented in Figure 2.

We particularly wanted to avoid a hierarchical pedagogic structure of “teaching to” HCWs. Peer-led programs have documented efficacy and may be as effective as professionally delivered formats [37, 38]. Peers also reduce expense, promote a sense of shared norms, enhance social support, and augment material with credible examples and personal experiences [39–41]. A downside of peer leaders is the need to recruit and train them. However, we have a history of successfully using scripted peer-led interventions, a format that minimizes training [35, 36].

Following the work-life check-in, the second component is an educational “lesson” with interactive activities to increase knowledge and practice skills related to health protection and promotion. For example, participants might learn what constitutes a serving size of fruit and vegetables, practice using simple ergonomic tools, or review a mnemonic for communication skills and role play its use. Meetings are once a month for six months. A listing of the TWH topics and activities in the six monthly meeting lessons is presented in Table 1.

**Table 1:** **Curriculum lessons, activitiesn and individual and team goals**

Session activities	Individual and team goals
Session 1: Fruits and vegetables	
• Barriers to eating fruits and vegetables	• Individual goal: tracking eating at least five servings each day or substituting for a sugary beverage each day
• Serving size quiz	• Team goal: pedometer challenge (team divided into two squads)
• Nutrition jeopardy	
Session 2: Back to healthy postures	
• Healthy spine (ship mast analogy)	• Individual goal: track neural spine position several times a day or attend relevant Oregon Home Care Commission (OHCC) class
• Practice with partner finding neutral spine	• Team goal: all do neutral spine task tracking
• Practice lifting postures	
• Tools to avoid back strain	
Session 3: Functional fitness	
• Core strengthen	• Individual goal: pair with teammate to exercise or core exercises everywhere tracking
• Sitting posture	• Team goal: repeat pedometer challenge
• Practice do anywhere core exercises	
• ABLE (abdomen, back and leg exercises) stickers	
• Benefits of exercise quiz	
Session 4: Take a load off with tools	
• Traps that lead to injuries	• Individual goal: watch video or attend OHCC class on tools
• Common injuries and how they occur	• Team goal: all assess hazards in consumer-employer’s home
• Low tech tool show-and-tell and practice	
• On tools	
Session 5: Communicate for hazard correction	
• Role play communicating with consumer-employer	• Individual goal: interview consume-employer about what makes good and bad days or use the PRAISE communication strategy with consumer-employer
• Learn PRAISE (Plan, Respect, Ask open-ended questions, I statements, Simply listen, Express understanding) mnemonic	• Team goal: all talk with consumer-employer about hazard
• Plan interaction	
• Role play interaction	
Session 6: Mental health	
• Practice total body relaxation	• Individual goal: choose relaxation or three blessing activity
• Three blessing activities	• Team goal: work on something from previous sessions
• Review of session topics and progress on goals	

We structured each lesson to end with verbalized selection and commitment to individual and team goals (Table 1). Follow-up would take place at the next month's lesson, where HCWs disclose progress and assess whether the team achieved their shared goal. In addition to building in social disclosure and reinforcing feedback [42–44], we planned team incentives to augment mutual accountability. For example, achieving 80% of team goals would be recognized with each team member receiving a COMPASS rain jacket at the end of the program.

**Figure 3:**
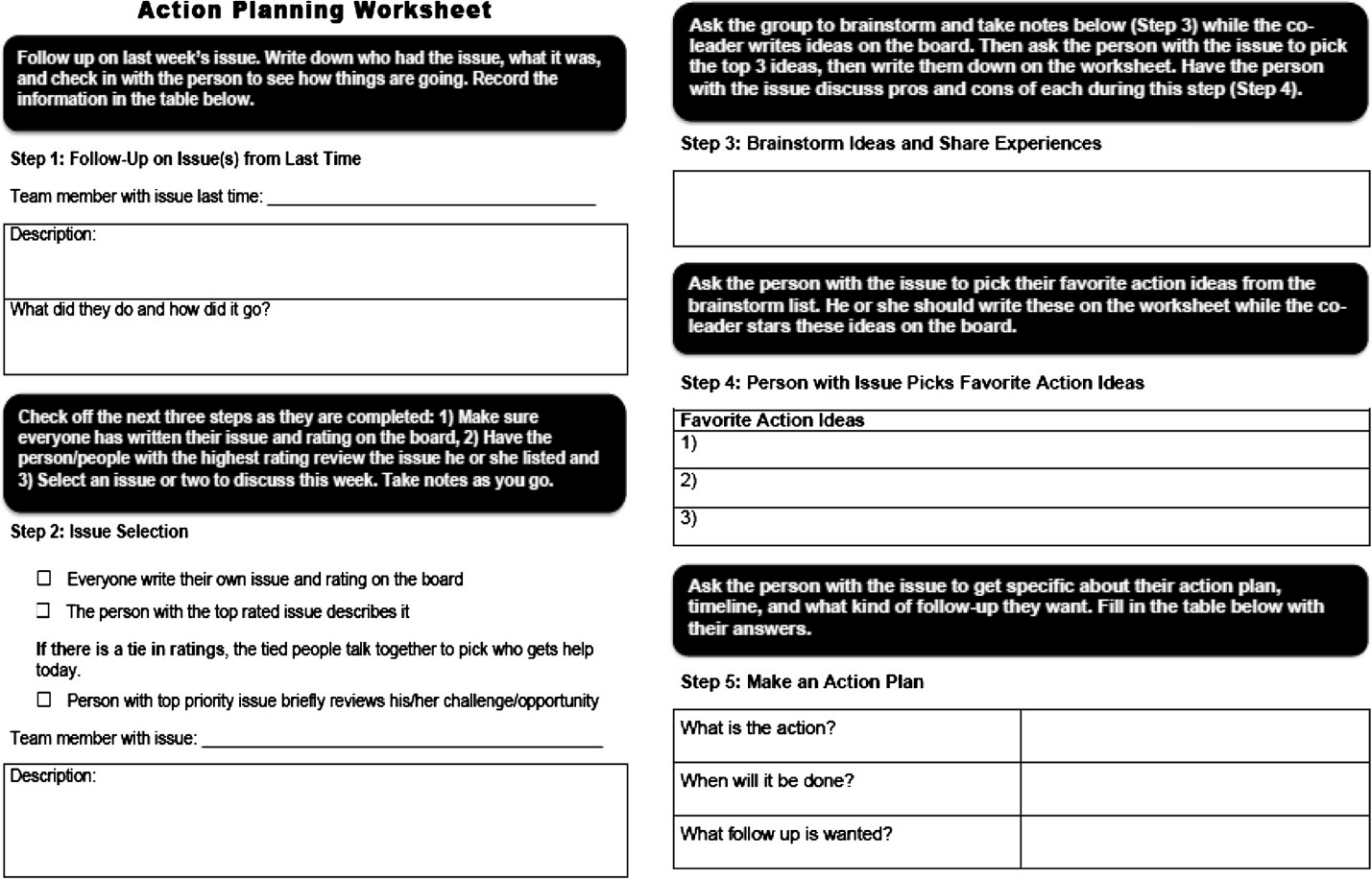
**Problem solving worksheet.**

Third, we provide a healthy meal, such as a vegetable soup and salad, at a break approximately halfway through the meeting, which is a time for members to socialize, while modeling healthy eating habits. The final meeting component is structured problem solving based on a successful process developed for family caregivers [45, 46] and intended to share knowledge on topics not addressed in the scripted lessons. Problem solving begins during the meal with problem nomination, where each individual writes a few words on a white board to describe a current problem, issue, challenge, or opportunity and rates it from A (urgent) to F (lowest priority) [33, 34]. The group selects one or two problems to address. During pilot testing, perhaps because of their occupational predisposition toward solving issues for their consumer-employers, workers tended to skip exploratory steps and jump straight into providing advice. Accordingly, we developed a worksheet to assist teams in following the steps of exploratory brainstorming and allowing the individual with the problem to select his/her own solution and action plan (Figure 3).

A review of studies indicated that social support from co-workers can have positive influences on affect, coping, absenteeism, job turnover and well-being [47]. In planning the meetings, roughly half the time was devoted to building social support, which is more than might be needed in a work structure that allows ongoing co-worker interactions. To further enhance HCWs sense of community and provide cues and tools for behavior change, intervention participants receive branded materials, such as a grocery bag, water bottle, and knee pads. The meeting closing includes a review of the session goals and a reflection on what each individual will remember from the session.

### Design for dissemination and wave-two recruitment

Existing TWH interventions were recently reviewed, and few have been used outside of their study setting [48]. We designed the COMPASS project to maximize dissemination potential, and data will go beyond participant behaviors and include indices of public health impact, such as economic outcomes [49].

Two dissemination strategies are planned, which will extend recruitment for the COMPASS trial. First, the Oregon Home Care Commission has existing paid training opportunities for HCWs. These 2- to 4-hour workshops are offered at various times and locations throughout the state. HCWs receive their hourly wage for attending each topic once per year. However, these largely didactic presentations lack follow-up on skill building and social support from bonded co-workers. To align with the pay for attending benefit, participants in COMPASS similarly are paid at the conclusion of each COMPASS session. If successful, the COMPASS curriculum could be inserted into the existing Home Care Commission offerings, with HCWs committing to a longitudinal sequence of COMPASS sessions. Investigators are working with the Oregon Home Care Commission to tailor the program to align with their format, with anticipated recruitment, using strategies similar to wave one but extended to other sites throughout Oregon, to begin later in 2014. Plans are to enroll four additional wave-two groups of Home Care Commission-COMPASS participants. Analysis plans for these groups are preliminary and await the initial wave findings. However, we anticipate comparing the process and outcome measures of wave one and two, and assessing the generality of a meditational model of effects developed with the original cohort.

Secondly, nationwide, the majority of HCWs are employed by private agencies [50]. Even in Oregon with its unique publicly funded model, approximately 40% of clients are served through a private agency. Study implementation methods will be collected and formatted into a manual of operation, all of which could be used within either public home care systems or private agency business structures. Discussions with private agencies and their local organizations are under way, and further plans for tailoring the program to specific agencies and implementing it within agencies await COMPASS trial outcomes.

## Methods

### Overview and timeline

The COMPASS trial is a cluster randomized controlled trial. Both intervention and the usual care control condition participants will complete health assessments and surveys prior to the intervention (month 0), at the end of intervention phase 1 (month 6) and at the end of intervention phase 2 (month 12). Additional funding is being sought for longitudinal assessments at 24 months.

### Participants: recruitment, randomization and retention

Our dispersed HCW population required a variety of recruitment approaches. The Portland, Oregon, area has approximately 3,500 unionized HCWs, and to include a population from a different locale, recruitment was expanded to include Eugene, Oregon, which has another estimated 1,000 HCWs. We developed a recruitment flyer that briefly described the study and emphasized three free health screenings over the course of a year and monetary compensation at $11 an hour (approximately the publicly-funded hourly rate at the time of study launch), with contact information for COMPASS staff. The flyers were distributed by research staff at existing Oregon Home Care Commission training meetings. Lastly, a short recruitment advertisement was sent via email and postal mail with the Service Employees International Union (SEIU) Local 503’s monthly newsletters.

Advertisements directed HCWs to call COMPASS staff, and an initial enrollment screening established eligibility and obtained verbal consent to participate. Inclusion criteria included working for at least one consumer-employer enrolled in a publicly funded program and their perceived ability to participate in a monthly meeting. We collected addresses, contact information, and preferred meeting times for use in clustering. If participants reported having substantial health concerns, such as heart-related issues, they were required to obtain a note from their physician before enrolling.

Eligible participants who provided verbal consent were assigned to a group based upon zip code, preferred meeting time, and enrollment order. To simplify coordination, workers were asked for a preference between two meeting times, either Saturday morning or Tuesday evening. Thus, clustering was not based on recruitment from a pre-existing organization but a virtual unit based on geographic location and ability to meet at a specific time and location. Once two clusters were populated, using a coin toss randomization technique, an outside researcher randomly assigned one group to control and the other to the intervention conditions.

*A priori* power analyses specified an enrollment target of 8 groups of 10 participants per condition. The average attrition rate across prior intervention studies with family caregivers was 20% [25], so we conservatively estimated attrition at 25% for each testing time point when designing the target sample size. To motivate retention, we plan lottery style drawings for participants who complete each health assessment wave. We also will have retention bonus pay (double hourly wages) for time spent during assessments after baseline. When two groups were recruited, they were randomized (one intervention and one control). Participants were informed of their condition assignment after their baseline data collection. All received the results of their health assessments, including a letter concerning desirable values, similar to that which might follow a typical visit to their health care provider.

### Control condition

Control participants are free to participate at their own discretion in usual training classes offered by the Oregon Home Care Commission.

### Data collection

Because there is no central “worksite”, participants are asked to report after a 3-hour fast to the SEIU Local 503 union hall in Portland or Eugene. The study protocol and all its procedures have approval from the Institutional Review Board at Oregon Health & Science University, and the initial act when participants report for testing was and will be completing written informed consent. Following that, simple physiological measures are obtained, a survey completed and a brief interview (during the 6- and 12-month assessment).

#### Quantitative

The HCW survey includes demographic, work-health history items and a battery of outcome and potential mediating measures. The survey emphasizes constructs with established reliability and validity. Behavioral items, unless otherwise noted, are anchored “in the past month.” Constructs and instruments used are presented in Table 2. The primary outcomes and purported mediators are indicated. Other variables are more distal outcomes, potential moderators of intervention effects or constructs added to align with other intervention trials being conducted at the Oregon Health Workforce Center of Excellence (http://www.cdc.gov/niosh/twh/centers.html).

**Table 2:** **Survey constructs and origins**

Primary outcomes	Purported mediators	Other variables
Fruit and vegetable servings [51]	Community of practice scale [52]	Demographics; health, work and habits history
Sugary drinks, fats and snacks [53]	Social support [54]	Job strain [55]
Self-reported physical activity scale [35]	Team cohesion [56]	Work stress [57]
Safety compliance [58]		Sleep deficiency [53, 59]
Self-reported tool use		Interpersonal conflict [60]
		Occupational Fatigue Inventory [61]
		Self-reported injury, workers’ compensation claims
		Bodily pain/musculoskeletal complaints [62, 63]
		Family work conflict [64]
		Family supportive supervisor behaviors [65]
		Positive and negative affect [66]
		Loneliness [67]
		Mood CES-D [68]
		Perceived stress [69]
		Stressful events [70, 71]
		Life satisfaction [72, 73]

The physiological assessment includes height, weight (Tanita scale; Model 310GS, Tanita Corporation of America, Inc., Arlington Heights, Illinois, USA) and blood pressure (OMRON Model HEM-907XL, Omron Healthcare, Inc., Lake Forest, Illinois, USA,). Fasting blood via routine finger sticks and capillary tube are analyzed for blood glucose, cholesterol, and triglyceride concentrations (Cholestech PA Analyzer, Hanson Medical Systems, Inc., Winter Park, Florida, USAA 6-minute walk test is used as an index of fitness [74]. A body mechanics assessment is performed using tasks: 1) picking up a bag of groceries from the trunk of a car, 2) picking up a box from the floor, 3) picking up a pen from the floor, and 4) buttoning the shirt of a client. Task demonstrations are videotaped and still shots identified at participant contact with object. Measurements of participant trunk flexion in degrees are obtained using a goniometer and compared to published risk ranges [75].

The consumer-employers of intervention and control participants also will be surveyed with an instrument that includes demographic questions, observations about safety behaviors and ratings regarding satisfaction with their HCW, using the 13-item Home Care Client Satisfaction Inventory [76]. Study participants deliver sealed survey packets to their consumer-employers that include information and the survey, as well as a pre-paid return addressed envelope.

#### Qualitative

Session observers take notes concerning the problem-solving topics discussed, items mentioned in reflections and other general observations. During the 6- and 12-month health assessments, semi-structured interviews will explore the process of attending sessions and behavior change. Open-ended questions will probe emergent themes, and those interviews are recorded for later transcription and analysis.

### Fidelity and adherence

A research staff member observes each COMPASS session, and the scripted curriculum provides a check list of session components to index fidelity and dose. The observer also takes notes as part of qualitative data collection.

### Power and sample size

Our wave one target sample was planned as 160 subjects (8 groups of 10 participants per condition), which is powered to detect changes in primary health promoting behavioral outcomes at the 6-month time point (0.85 power), based on effect sizes concerning specific eating behaviors (frequency of sugary drink and sugary snack consumption) from another population of lone workers [77] and an estimated intraclass correlation of 0.02 for health behaviors, based on a meta-analysis of intraclass correlations in studies of health behaviors [78]. The design also provides acceptable (but not exceptional) power to detect maintenance effects at the 12-month time point (0.74 power). Based on the original wave's eight intervention and eight control groups, the study should have moderate power (0.72) to detect mediated effects at the 6-month time point where both of the paths to and from the purported mediator exhibit medium effect sizes (that is, a = 0.3 and b = 0.3). The wave two mediational model will be compared with the wave one findings. While power will be limited given the smaller sample size of 40 participants (four groups) in the second translational wave, we will compare effect sizes in each pathway as they relate to the model of mechanisms developed with the original cohort.

### Data analysis

#### Quantitative

In general analyses we will use SPSS (SPSS, Chicago, IL, USA) and M-Plus (Muthén and Muthén, Los Angeles, California, USA) for structural equation modeling. Survey instrument assessment will begin by confirming predicted item constructs, augmented with exploratory factor analysis, to establish reliable summary scales with maximum internal consistency.

Our primary hypothesis is that the intervention will be more effective than a usual treatment control condition for increasing safety and health behaviors. To test these hypotheses, we will evaluate between-group differences using an intent-to-treat approach and generalized estimating equations with participants nested in groups. We will begin by computing interaction contrasts on pre/post-intervention differences across groups. Intervention group membership will be the between-subjects factor, and pre/post-intervention assessment scores on primary outcomes will be the within-subjects factor. The interaction contrast approach will allow efficiently comparing the two groups on each dependent measure with a focused one degree of freedom hypothesis test; similar analyses will be repeated for the follow-up assessment time point.

We will also test for group differences in secondary outcomes, such as injuries and health measures. Discrete-scaled and rare secondary outcomes (for example, injuries) will be analyzed with Poisson or zero-inflated Poisson regression models. In models for discrete-scaled outcomes, we will use baseline values as a pre-treatment covariate to account for existing individual differences and boost statistical power to detect intervention effects.

Using mediation analysis to deconstruct the action and conceptual theory components has been a feature of our programs [79–81]. For continuous outcomes, structural equation modeling will be used to evaluate relations among variables using model-fit indices. For binary or ordinal outcomes, each mediator’s contribution to predicting group states for outcomes will be conducted using logistic regression analysis. Cross-sectional and longitudinal models will be developed to identify the strength of the relation of purported model constructs to outcome variables, and model-fit indices calculated. The multilevel capabilities of latent growth modeling make possible construction, estimation, and testing of a variety of complex models utilizing hierarchically structured data, such as ours. Each potential mediating variable will be evaluated in separate models, followed by multiple mediator models. Longitudinal mediation effects will be assessed in the latent growth modeling framework. These methods also allow for identification of subgroups for which the program has a differential effect. Variables coding these subgroups are moderator variables, and they usually are equivalent to interaction effects. Models with mediators and moderators allow simultaneous assessment of how a program works (mediation) and whether or not the program works differentially for certain participants (moderation). For example, a common result in prevention research is that effects vary according to pre-existing risk. This type of mediation modeling can provide information about subgroups experiencing large effects and others for which it is ineffective or even counter-productive.

For the initial randomized control trial wave, all participant data will be used, and group assignment will be represented by a dummy code variable (that is, intervention = 1, control = 0), using the pretest measures as predictors of the corresponding post-test outcomes and medicating variables, with the dummy code variable accounting for group differences at baseline. Once that mediation model is developed and we have completed the dissemination wave, we will assess and measure the magnitude of each pathway with data from participants who complete the adapted Home Care Commission-COMPASS program. While we do not expect that pathways in those latter analyses will be statistically significant, given the small sample size, we will compare effect sizes in each pathway as they relate to path effects in the original cohort.

We will determine the intervention’s economic impact by measuring and computing intervention costs and contrasting them with estimated outcome-related savings. Our survey measures include potential cost saving outcomes, such as self-reported lost work time due to injuries and illnesses, absenteeism, and turnover intentions. We will also collect expanded economic-related data from agencies and organizations that “own” the data for our population of HCWs. We plan to work with the SEIU health care trusts to obtain health care cost data from health care providers for HCWs participating in the study. We will request these expanded workers compensation and health care cost data for participants in both arms of the study retrospectively (36 months prior to enrollment) and prospectively (12 months post-enrollment).

#### Qualitative

Thematic content analysis will begin with reading interview transcripts and notes to identify patterns, commonalities, and idiosyncrasies. Barriers and enhancements to program impact will be sought within the dynamic contexts of actual experience. Construction of themes and interpretations [82–84], rather than bounding interpretation with a pre-specified list of categories for coding, avoids the substantive bias inherent in *a priori* categorization. Instead, we will use an adaption of the more inductive constant-comparative method [85, 86]. Following identification of themes, the data set will be subjected to micro-review in a search for both confirming and disconfirming data. Quotations will be selected to exemplify and elaborate themes, to support findings, and to illuminate contexts, promoting reader understanding and facilitating generalization to other occupational settings. Gathering qualitative findings also will allow developing case studies [87]. We anticipate that these case studies may be useful when sharing findings with the Oregon Home Care Commission and others.

### Ethical issues

All study procedures were reviewed and approved by the Oregon Health & Sciences University Institutional Review Board (IRB00005473), with attention to informing potential participants that their enrollment will in no way affect their relationship with their union, consumer-employers, or Oregon Health & Sciences University. Institutional Review Board approval includes a data safety and monitoring plan, including the Principal Investigator’s close monitoring and prompt reporting of any adverse events.

## Discussion

The COMPASS study is a cluster randomized controlled trial to improve the health and safety of HCWs. Its design is theory-based and structured to meet the needs of these low-wage, isolated workers. Mediation analysis will assist in confirming whether the program impacts purported mediating variables and validates its conceptual underpinnings. The program has several unique components. First, it is held away from the job as, unlike typical employees, HCWs lack a shared worksite. Second is the explicit attention to building a community of practice and providing social support for these lone workers. The scripted, peer-led format has worked in other settings [35, 36], and for HCWs it may further enhance relevance and social support. In addition, having both individual and team level goals operating simultaneously may augment behavior change [31, 32].

We recognized that HCWs have busy home lives, often also providing care for family members, and that they may change consumer-employers during the program, making attendance difficult. Sessions are being held at locations and times to facilitate participation, and strategies are in place to minimize attrition. As with Oregon Home Care Commission classes, HCWs are paid for attending each session, and an escalating reimbursement structure accompanies data collection activities. In formatting manuals and workbooks, we needed a balance between a scripted format to maintain fidelity across groups and enough flexibility to build ownership and allow unscripted interactions that would enhance social support. We will observe all sessions, record adherence to the format, and document any deviations.

The lessons have a balance of safety and health promotion topics. Review of the literature identified only 17 published TWH programs that addressed both safety and health promotion in their intervention and assessment methods, and most lacked a balanced focus [48]. Simultaneously working on health protection and promotion may have advantages. Recent comparison of concurrent versus sequential behavior interventions indicates that addressing behaviors simultaneously is as effective and may be synergistic [88–90].

Some of the study’s unique features also relate to its limitations. The unionization of Oregon HCWs provides a large population that can be configured in control and intervention groups for balanced randomization to study conditions. However, participants are from population centers and may not be representative of HCWs from more rural areas. Although the assessment measures are robust, in part to harmonize COMPASS with other studies of the Oregon Healthy Workforce Center for cross-project analyses, many are self-report, and our connections with intervention participants may lead to social desirability bias. In fact, the original description of altered behaviors because of being studied was among workers at the Western Electric Company’s Hawthorne Plant [91, 92]. However, although intuitively a potential factor, when examined, social desirability has minimal impact on intervention findings [93], and we will minimize any potential effect with attention to item wording, confidential survey completion and assessing those issues in the qualitative assessments.

The COMPASS study is designed to assess the efficacy of a TWH program for HCWs. Its strategy and methods demonstrates how a worker health protection and promotion intervention can be aligned with the unique needs of a particular worker group. If efficacy is established, a means for translation could allow enhancing the well-being of a growing workforce responsible for caring for frail and vulnerable members of the community.

### Trial status

Two pilot groups of HCWs informed the intervention meeting’s structure and session content, using a just-in-time format. That is, observations and feedback from each pilot group session informed the curriculum development for the next meeting. At the time of this manuscript submission, participants for an initial wave of the trial have been recruited and are engaged in the study protocol. Their baseline data is being assembled and cleaned; no baseline analyses have occurred. Wave two participant recruitment for the planned four HCW groups for the Home Care Commission-COMPASS program will begin late in 2014.

## Abbreviations

COMPASS: COMmunity of Practice And Safety Support
HCW: home care worker
NIOSH: National Institute of Occupational Safety and Health
SEIU: Service Employees International Union
TWH: Total Worker Health™.
